# Core-Shell Structured Magnetic Carboxymethyl Cellulose-Based Hydrogel Nanosorbents for Effective Adsorption of Methylene Blue from Aqueous Solution

**DOI:** 10.3390/polym13183054

**Published:** 2021-09-10

**Authors:** Yiming Zhou, Te Li, Juanli Shen, Yu Meng, Shuhua Tong, Qingfang Guan, Xinxing Xia

**Affiliations:** 1 College of Textile Science and Engineering, International Institute of Silk, Zhejiang Sci-Tech University, Hangzhou 310018, China; zymllm@163.com (Y.Z.); zstu_lt@163.com (T.L.); shenjuanli2021@163.com (J.S.); 2 Zhejiang Jinchang Special Paper Co., Ltd., Quzhou 324400, China; mengdreamyu@126.com (Y.M.); tong13857034900@163.com (S.T.); 3 Anning Decco Fine Chemical Co., Ltd., Kunming 650301, China; gqfang123@163.com

**Keywords:** nanosorbents, magnetic nanocomposite, dye adsorption, hydrogel, methylene blue

## Abstract

This article reports effective removal of methylene blue (MB) dyes from aqueous solutions using a novel magnetic polymer nanocomposite. The core-shell structured nanosorbents was fabricated via coating Fe_3_O_4_ nanoparticles with a layer of hydrogel material, that synthesized by carboxymethyl cellulose cross-linked with poly(acrylic acid-co-acrylamide). Some physico-chemical properties of the nanosorbents were characterized by various testing methods. The nanosorbent could be easily separated from aqueous solutions by an external magnetic field and the mass fraction of outer hydrogel shell was 20.3 wt%. The adsorption performance was investigated as the effects of solution pH, adsorbent content, initial dye concentration, and contact time. The maximum adsorption capacity was obtained at neutral pH of 7 with a sorbent dose of 1.5 g L^−1^. The experimental data of MB adsorption were fit to Langmuir isotherm model and Pseudo-second-order kinetic model with maximum adsorption of 34.3 mg g^−1^. XPS technique was applied to study the mechanism of adsorption, electrostatic attraction and physically adsorption may control the adsorption behavior of the composite nanosorbents. In addition, a good reusability of 83.5% MB recovering with adsorption capacity decreasing by 16.5% over five cycles of sorption/desorption was observed.

## 1. Introduction

Synthetic organic dyes are one of the main factors that cause environmental contamination, and the problem has become more and more serious with the development of dyeing industries. In fact, over 10% of the annual produced 7 × 10^5^ tons dyes are effluents coming from coloring textiles, printing, varnishes, and cosmetics, as well as dye manufacturing and plastic industries [1,2,3]. Dyes can be extremely dangerous to human health due to their complex composition, high toxicity, high solubility in water, and poor degradability [4,5]. Therefore, it is imperative to devise effective remediation of dyes from wastewater [6,7].

Numerous existing technologies including ultrafiltration [8], ion exchange [9], photocatalysis [10], membrane separation [11], and adsorption [12] have already been used in wastewater treatment. Adsorption, as a promising technology, has received increasing attention owing to its high efficiency, easy operation, little impact, low cost, and excellent recycling performance [13,14]. However, the difficulty of collecting the adsorbents from the treated wastewater after adsorption restricts its use in practical treatment. Recently, a new type of magnetic composite has been developed, which offers an advantage due to the easy and rapid separation by means of a magnet. Besides, when the adsorbents have sizes in nanometer range, the magnetic interactions between particles can be reduced, bringing a superparamagnetic behavior that can diminish the particle aggregation while preserve the suspension stability [15,16]. For this reason, magnetic nanosorbents have been designed and successfully applied for the removal of dye [17,18]. It is expected that the magnetic composites will receive increasing attention in the field of environmental remediation.

Carboxymethyl cellulose (CMC), a type of linear biopolymer, has been extensively utilized in pollution control owing to its outstanding characteristics of abundant, cheap, and biodegradable [19]. It has good adsorption capacity and high affinity toward environmental pollutants because of the existence of functional groups (hydroxyl and carboxyl) to form complex by interaction with multivalent ions or cationic polymer [20,21]. CMC-related adsorbents are usually prepared in the form of hydrogels, hydrogels are high-performance materials with three-dimensional networks made by chemical and/or physical cross-linking [22,23]. CMC-based hydrogel can provide the reaction and coordination sites for dyes through a series of electrostatic interaction and coordinated complexation processes, that making it more suitable for use in dyes adsorption [24,25]. However, incorporation of magnetic nanoparticles within the CMC-based hydrogel to form a nano-composite for adsorption of dye pollution has been rarely explored. Compared to micro- and macro-composites, nanocomposites provide more improved or even novel properties. Nanohydrogel with inorganic cores shows extraordinary change in physico-chemical properties along with active interface performance, ease of separation, and higher surface area as compared to parental hydrogel matrix [4,26].

Nowadays, nanosized Fe_3_O_4_ have been widely used for the production of magnetic nanosorbents due to excellent properties such as good compatibility, nanometer size, and magnetic property for easy separation from the aqueous solution [27,28,29]. Under the guidance of the development concept, in present study, core-shell structured nanocomposites that combine Fe_3_O_4_ cores and CMC-based hydrogel shells were designed. The magnetic hydrogel nanocomposites were synthesized via the combination of graft co-polymerization and chemical co-precipitation methods. The obtained nanocomposite was used for the removal of MB dyes. The properties of the nanosorbent were characterized by various techniques. The adsorption behavior was evaluated under a series of conditions, the adsorption mechanism was discussed according to the adsorption isotherms and kinetics.

## 2. Experimental

### 2.1. Materials and Reagents

Powder of FeCl_2_·4H_2_O and FeCl_3_·6H_2_O were purchased from Zhanyun Chemical Co. Ltd. (Shanghai, China). Carboxymethyl cellulose sodium (CMCNa), ammonium persulfate (APS), acrylic acid (AA), acrylamide (AM), *N*,*N*-Dimethylformamide (MBA), methylene Blue (MB) were obtained from Aladdin Shanghai Chemical Co., Ltd. All other chemicals and reagents were of analytical grade. A total 1000 mg L^−1^ stock solution of MB was prepared by dissolving appropriate amount of dye in 1 L of NaNO_3_ electrolyte solution (0.01 M) and diluted for further batch experiments. Deionized water was used throughout the experiments.

### 2.2. Preparation of Magnetic CMC and Magnetic Hydrogel Nanocomposite

For fabrication of magnetic CMC nanoparticles (mag-CMC), 1.32 g FeCl_2_·4H_2_O and 1.80 g FeCl_3_·6H_2_O was dissolved to prepare a 100 mL iron-containing aqueous solution. Then the solution was heated to 50 °C after 1.5 mol L^−^^1^ NaOH (20 mL) was dropwisely added. After continuous stirring for 30 min, 100 mL of 15 mmol L^−1^ CMCNa solution was added into the reaction and the resulting mixture was heated to 70 °C and stirred for 3 h under continuous N_2_ atmosphere. Finally, the resulted mag-CMC was isolated with the help of a magnet field and washed several times with water until the washwater became neutral, and vacuum-dried for further use.

For preparation of magnetic CMC-based hydrogel nanoparticles (mag-hydrogel), CMC-g-p(AA-co-AM) hydrogel was synthesized outside the magnetic cores through free radical polymerization method by using MBA as a crosslinking agent and APS as an initiator. Briefly, on the basis of mag-CMC preparation, after adding CMCNa for 30 min, 25 mg MBA, 25 mg APS, 0.5 g AM and 0.5 mL AA were added to the reaction mixture. The above mixture was heated to 70 °C and continuously stirred for 3 h under N_2_ atmosphere. Finally, the formed mag-hydrogel was collected, washed thoroughly, and vacuum-dried for further use.

In order to alter the mass fraction of the hydrogel materials out of the magnetic cores, the operation of polymerization was carried out again outside the surface of mag-hydrogel. Briefly, certain amount of mag-hydrogel was weighted and added into 15 mmol L^−1^ CMCNa solution (100 mL). The following operations and reagents additions were consistent with the fabrication of mag-hydrogel. The recoated mag-hydrogel obtained was referred to as Recoated-mag-hydrogel.

### 2.3. Characterization Methods

X-ray diffraction (XRD, ARL XTRA, Westborough, MA, USA) of the samples were done at radiation of 1.542 Å wavelength (Cu Kα) operated at 40 mA and 45 kV. The determination of grafting of CMC-g-p(AA-co-AM) hydrogel on magnetic cores within the nanocomposite were analyzed on a Nicolet 5700 (Thermo Nicolet, Carlsbad, CA, USA) fourier transform infrared (FTIR) spectrometer using KBr pallet method in spectral range of 4000–400 cm^−1^. The specific surface area measured by the method of N_2_ gas adsorption was obtained by Brunauer-Emmett-Teller (BET, 3H-2000PS1, CHN) method. Magnetic measurements were investigated using a Quantum Design vibrating sample magnetometer (VSM) in an applied field from −10,000 Oe to 10,000 Oe. Thermogravimetric analysis (TGA) of magnetic nanocomposites were studied by using TGA analyzer (PYRIS 1, USA), 5.0–10.0 mg of the sample was heated from 30 °C to 700 °C with the heating rate of 10 °C min^−1^ under N_2_ flow of 10 cm^3^ min^−1^. The zeta potentials in the pH range from 2.0 to 9.0 were conducted by Zetasizer Nano ZS90 Analyzer (Malvern, UK) at room temperature. Zero point charge (pH_zpc_) were performed by interpolating the zeta potential data to zero. The binding energies of the nanosorbents before and after MB adsorption were studied using X-ray photoelectron spectroscopy (XPS, PHI5000 Versaprobe, Chanhassen, MN, USA).

### 2.4. Adsorption Experiments

A series of experiments for the adsorption of MB solution were performed using a thermostatic shaker bath operated at 100 rpm at 25 °C. In the typical adsorption experiments, magnetic nanosorbents were weighted and added to 100 mL MB solutions with suitable concentration, and a contact time of 4 h was sufficient to reach adsorption equilibrium. Then suspensions were taken out and filtered through a 0.45 μm millipore membrane to remove the adsorbent. The MB concentration remained in the solutions were determined on a UV/Vis spectrophotometer (Cary 60, Agilent, Santa Clara, CA, USA) at 665 nm as λ_max_ of MB [30]. The experimental adsorption data for the removal of MB was calculated according to the Equation (1):

(1) $$\%\;adsorption\;capacity=\frac{\left(C_{0}-C_{e}\right)}{C_{0}}\times 100\%$$

where *C*_0_ is the initial concentration and *C_e_* (mg L^−1^) is the equilibrium concentration of dye solution, respectively. Effect of solution pH on MB adsorption was evaluated in the pH range of 4.0–10.0 and the pH was adjusted by using 0.1 M HCl and 0.1 M NaOH solutions. Influence of adsorbent dosage on MB adsorption was investigated over the adsorbent amount range of 0.25–3.0 g L^−1^ at pre-optimized conditions.

For adsorption isotherm experiments, optimized amount of magnetic nanosorbents (150 mg) was added into a series of 100 mL MB solutions with different concentrations varying from 5 to 200 mg L^−1^ at pH 7.0 for 4 h. The experimental adsorption data at equilibrium was calculated according to the Equation (2):

(2) $$q_{e}=\frac{\left(C_{0}-C_{e}\right)}{m}\times V$$

where *q_e_* (mg g^−1^) is the adsorption of MB per unit mass of adsorbent at equilibrium, *m* (g) is the amount of the adsorbents used and *V* (L) is the MB solution volume.

Adsorption kinetic studies were carried out at initial MB concentration of 75 mg L^−1^. In kinetics studies, 150 mg of magnetic nanosorbents was added to 100 mL 75 mg L^−1^ MB solution at pH 7.0. At predetermined time intervals, about 2 mL mixture was collected and filtered for the analysis of residual MB concentration in solutions. The adsorption capacity of MB at different time intervals were calculated according to the Equation (3):

(3) $$q_{t}=\frac{\left(C_{0}-C_{t}\right)}{m}\times V$$

where *q_t_* (mg g^−1^) is adsorption of MB per unit mass of adsorbent at time *t* (min) and *C_t_* (mg L^−1^) is concentration of MB solution at time *t* (min).

### 2.5. Reusability Experiments

Consecutive regeneration cycles were used to determine the reusability of magnetic nanosorbents. For these experiments, 150 mg adsorbent was added to 100 mL of 75 mg L^−1^ MB solution at pH 7.0. After adsorption for 4 h, the MB-loaded adsorbent was separated with a magnet and the desorption operation was carried out by dispersing the adsorbent into 100 mL of 0.1 M HCl solution at 150 rpm for 120 min at room temperature. Regenerated adsorbents were again used for the adsorption of MB at optimized experimental conditions. The adsorption-desorption cycles were repeated 5 times by using the same adsorbents.

## 3. Results and Discussion

### 3.1. Characterization of Magnetic Materials

**Figure 1:**
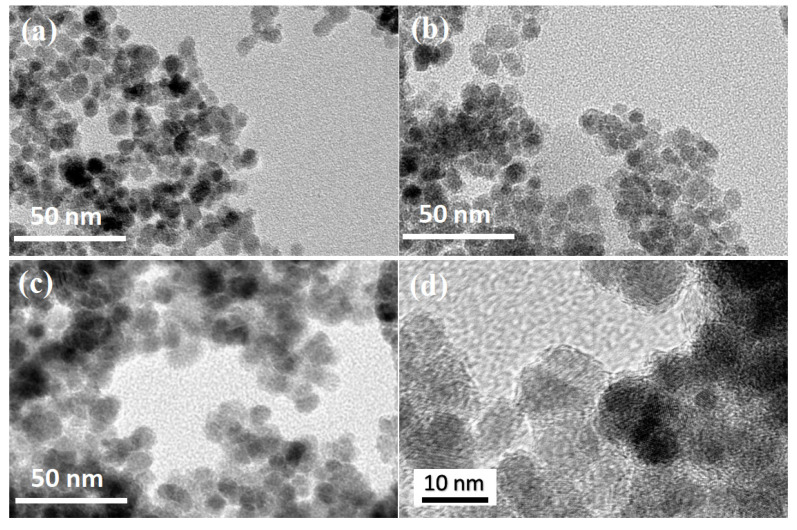
TEM morphologies of (**a**) Fe_3_O_4_; (**b**) mag-hydrogel; (**c**) recoated-mag-hydrogel; and (**d**) recoated-mag-hydrogel in a larger version.

TEM images (Figure 1) of uncoated Fe_3_O_4_ nanoparticles and magnetic nanocomposites are shown in Figure 1. It is obvious that Fe_3_O_4_ was spherical with a smooth surface, and the mag-hydrogel surface was relatively rough. Besides, the diameter of mag-hydrogel, recoated-mag-hydrogel were found to be higher than that of uncoated Fe_3_O_4_. The larger TEM image of recoated-mag-hydrogel in Figure 1d clearly showed that Fe_3_O_4_-core was coated with translucent hydrogel shell. The TEM results confirmed the successful synthesis of the core-shell structured nanosorbents.

**Figure 2:**
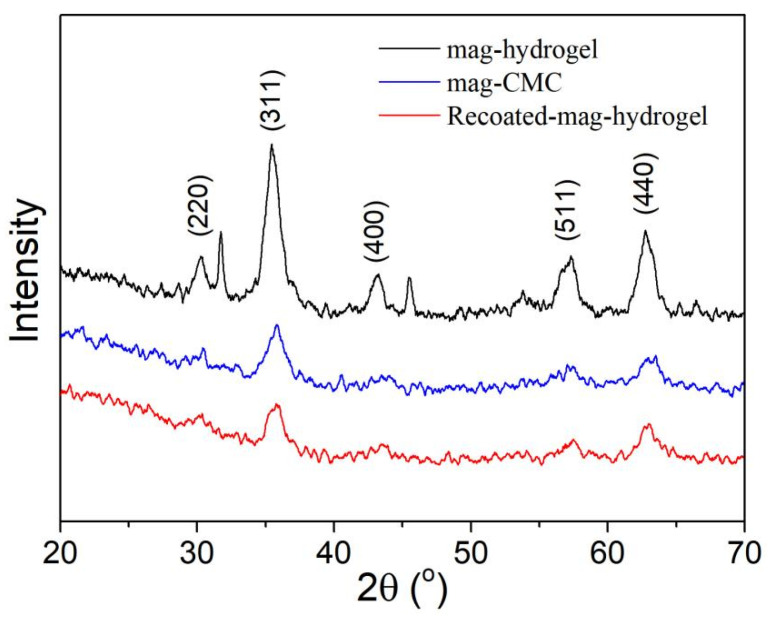
XRD patterns of magnetic nanocomposites.

Figure 2 displays the XRD patterns of the obtained magnetic nanocomposites. Based on data of the JCPDS card for Fe_3_O_4_ (JCPDS No. 19–0629) [31], the diffraction peaks at (220), (311), (400), (511), and (440) planes can be indexed to be cubic Fe_3_O_4_ phase. These characteristic peaks were observed in mag-CMC and mag-hydrogel, which proved that Fe_3_O_4_ was contained in these synthesized nanocomposite. It can be concluded that the magnetic nanocomposites in present study have the magnetic core of Fe_3_O_4_. However, because of increasing mass fraction and different network structure of the non-magnetic polymeric shell, the Fe_3_O_4_ cores diffraction intensity were slightly reduced.

**Figure 3:**
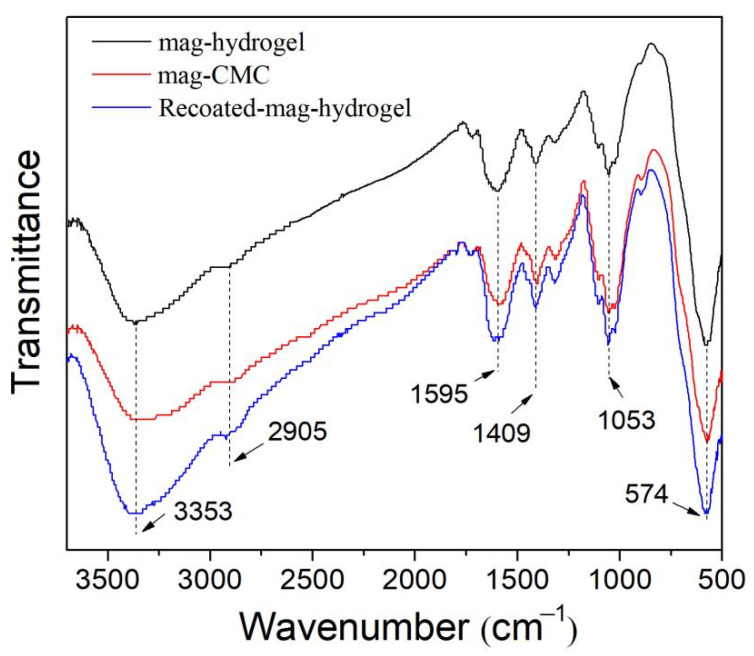
FTIR spectra of magnetic nanocomposites.

FTIR spectra of magnetic nanosorbents are illustrated in Figure 3. The sharp bands at 574 cm^−1^ of all the samples were ascribed to Fe−O vibrations in Fe_3_O_4_ phase [1]. Besides, the peaks appearing at 3353 and 2905 cm^−1^ were the stretching vibration peaks of −OH and C–H bonds, respectively. Meanwhile, 1409 cm^−1^ was the stretching vibration in skeletal C=C in the aromatic rings. Besides, the characteristic peaks of C=O at 1595 cm^−1^, and *β*-1,4-glycosidic bond at 1053 cm^−1^ were also observed. These peaks can be attributed to the cellulose structure in CMC polymer [19]. The spectra of mag-hydrogel was very similar to that of mag-CMC, no significant characteristic peak ascribed to AA or AM was observed. These may be resulted from the relatively low content of AA and AM in synthesized hydrogel shell.

**Figure 4:**
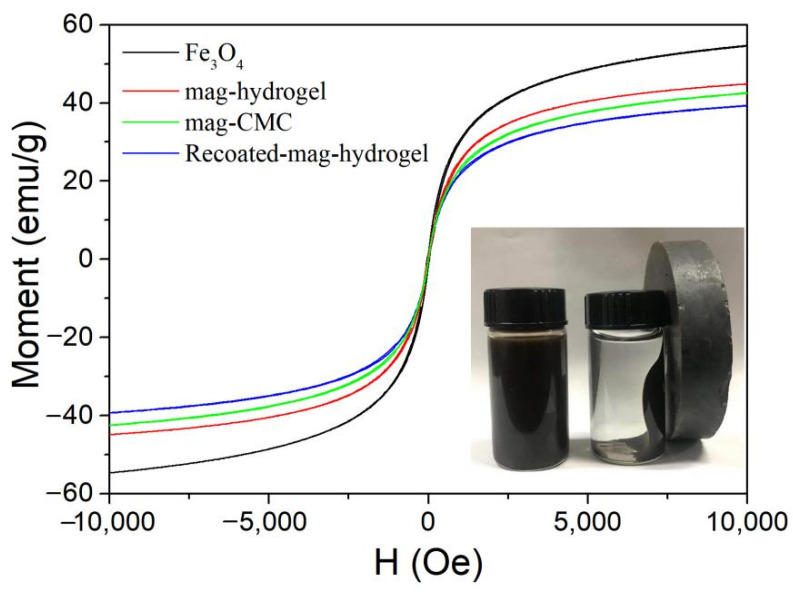
Magnetization curve of Fe_3_O_4_ and magnetic nanocomposites.

Figure 4 shows the magnetization curves of synthesized Fe_3_O_4_ magnetic nanocomposites. The saturation magnetization (*M*s) of mag-CMC, mag-hydrogel, and Recoated-mag-hydrogel were found to be 42.6 emu g^−1^, 44.8 emu g^−1^, and 39.4 emu g^−1^, respectively, lower than that of Fe_3_O_4_ at around 54.7 emu g^−1^, which can be attributed to the non-magnetic polymer coating (CMC or hydrogel) out of the Fe_3_O_4_ cores. The decreasing trend of these magnetic samples in *M*s were consistent with the results of XRD. The inset performance in Figure 4 affirmed that even Recoated-mag-hydrogel at lowest *M*s of 39.4 emu g^−1^ could also be easily recovered from the waste solutions by the application of an external magnetic field.

**Figure 5:**
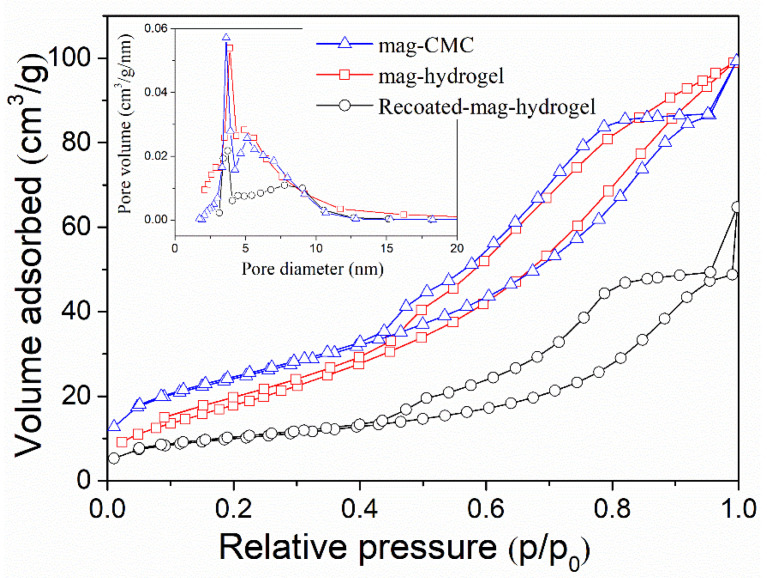
N_2_ adsorption-desorption isotherm and pore size distribution curves of magnetic nanocomposites.

Figure 5 analyzes the specific surface area of magnetic nanocomposites by BET results. For mag-hydrogel, the BJH pore size was 5.5 nm and the specific surface area was 73.5 m^2^ g^−1^, compared to 5.2 nm and 87.2 m^2^ g^−1^ of mag-CMC, respectively. Recoated-mag-hydrogel has higher pore size of 6.1 nm and lower surface area of 35.3 m^2^ g^−1^. These results revealed that the specific surface area of the core-shell nanocomposites decreased with the increasing thickness of the outer polymeric shell. Besides, because of the porous inner structure of hydrogel, the nanosorbents with hydrogel coating has larger pore sizes, which is beneficial to the adsorption performance.

**Figure 6:**
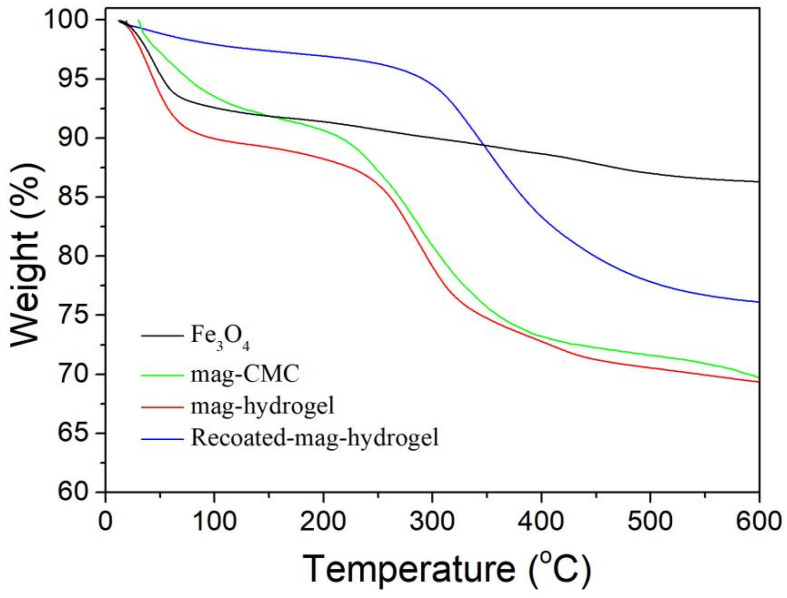
Thermogravimetric curves of Fe_3_O_4_ and magnetic nanocomposites.

TGA can be employed to quantitatively assay the amount of substrate-attached material that remained on the surface, the TG results are shown in Figure 6. Gradual degradation within 20–180 °C resulted from the removal of free and combined water. The weight loss from 200 to 350 °C corresponded to the decarboxylic process of CMC, AA, or AM molecules. At higher than 350 °C, the weight loss was related to the breaking of C–O bonds in the CMC or CMC-g-p(AA-co-AM) structure. When all the samples were heated up to 500 °C, no distinct difference in weight loss was observed, demonstrated that only iron oxide was present in this step. On the basis of TG curves, the mass fraction of outer CMC or hydrogel shell were 17.6 wt%, 17.5 wt%, and 20.3 wt% for mag-CMC, mag-hydrogel, and Recoated-mag-hydrogel, respectively.

**Figure 7:**
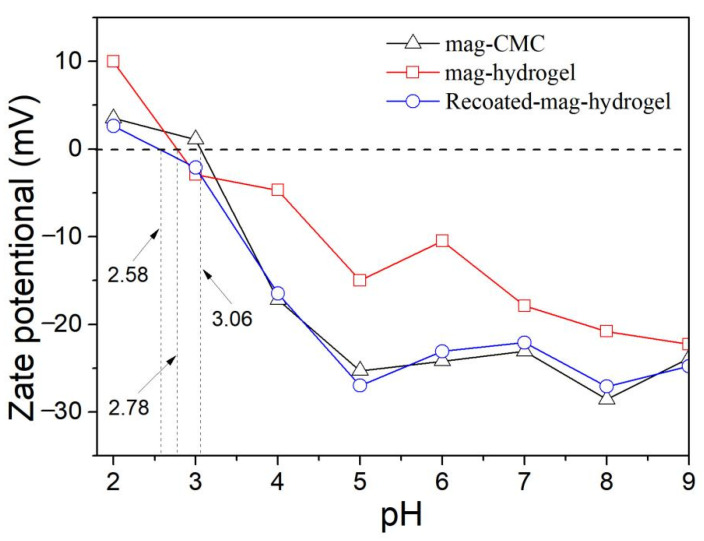
Zeta potentials of magnetic nanocomposites at various solution pH values.

Figure 7 depicts the zeta potential data of as-prepared magnetic nanocomposites. The pH_zpc_ of mag-CMC and mag-hydrogel were identified to be 3.1 and 2.8, respectively. Since the magnetic nanocomposites in present study have the magnetic core of Fe_3_O_4_, it is worth noting that the pH_zpc_ of mag-CMC or mag-hydrogel were lower than that of Fe_3_O_4_, ranging from 6.0 to 7.0 as reported in the previous studies [32], suggesting the successful covering of CMC or hydrogel outside the Fe_3_O_4_ cores. For mag-CMC, the pH_zpc_ at 3.1 was also lower than that of CMC at pKa value of 3.2–4.3 [33], which could be tentatively interpreted as resulting from the different synthesis condition. At present work, CMC molecules were facilely adhered to the Fe_3_O_4_ surface by electrostatic self-assembly approach. The specific active FeOH groups on the surface of Fe_3_O_4_ could effectively be replaced by the carboxyl groups of CMC, resulting in composite samples with reduced zeta potential [34,35]. Besides, the pH_zpc_ of mag-hydrogel (2.8) and Recoated mag-hydrogel (2.6) was lower than that of mag-CMC (3.1), because the surface charge was also dependent on the ionization degree and 3D structure in CMC-g-p(AA-co-AM) hydrogel [34].

### 3.2. Effect of Solution pH

**Figure 8:**
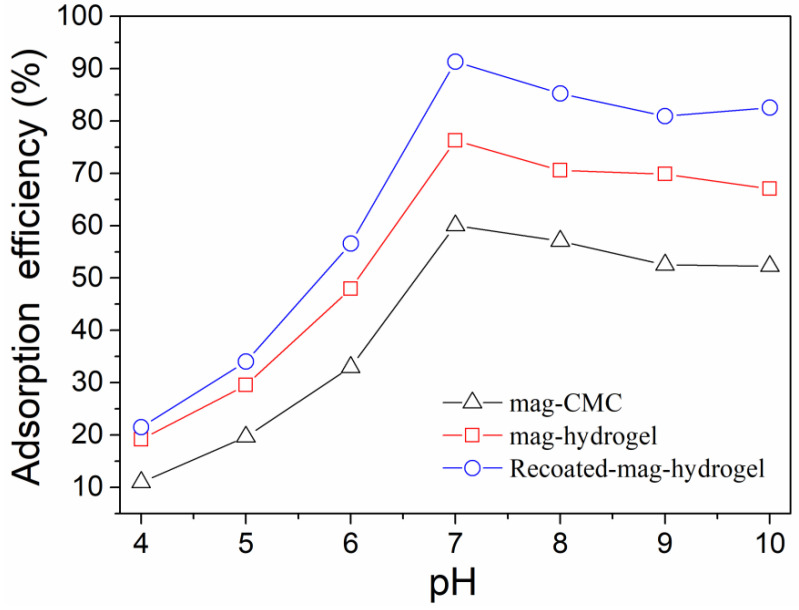
Effect of solution pH on MB adsorption onto magnetic nanocomposites.

Effect of solution pH on MB adsorption efficiency by using present magnetic nanocomposite has been studied. As shown in Figure 8, for all the absorbents, in the pH range of 4.0–7.0, the absorption efficiency of MB increased with increasing pH value. As mentioned above, the mag-CMC or mag-hydrogel nanosorbent had a pH_zpc_ value of 2.5–3.0. Therefore, within the measured pH range (Ph > pH_zpc_), the surface of these adsorbents were negatively charged. In addition, the surface would become more negatively charged with increasing pH because of the decreasing concentration of H^+^ ions in MB solution, which was more favorable to the strengthening of the chemical interaction between the cationic dye and the deprotonated surface sites [36]. However, at pH of 7.0–10.0, the adsorption efficiency of all the adsorbents slightly decreased, the reason might be that most of the adsorption sites on the surface of the adsorbents were combined and occupied by the large amount of OH^-^ ions in that pH solution. Higher adsorption efficiency of MB at 91.3% was observed by using Recoated-mag-hydrogel nanosorbent. Since CMC, AA, and AM were all anionic polymers with sufficient negatively charged binding sites, MB was a cationic dye that can bind easily to the negatively charged sites in anionic adsorbents. Therefore, CMC-g-p(AA-co-AM) based hydrogel had a very good affinity for combining of MB molecules. Moreover, higher mass fraction of hydrogel layer would increase the dye binding sites of the core-shell magnetic nanosorbent.

### 3.3. Effect of Adsorbent Content

**Figure 9:**
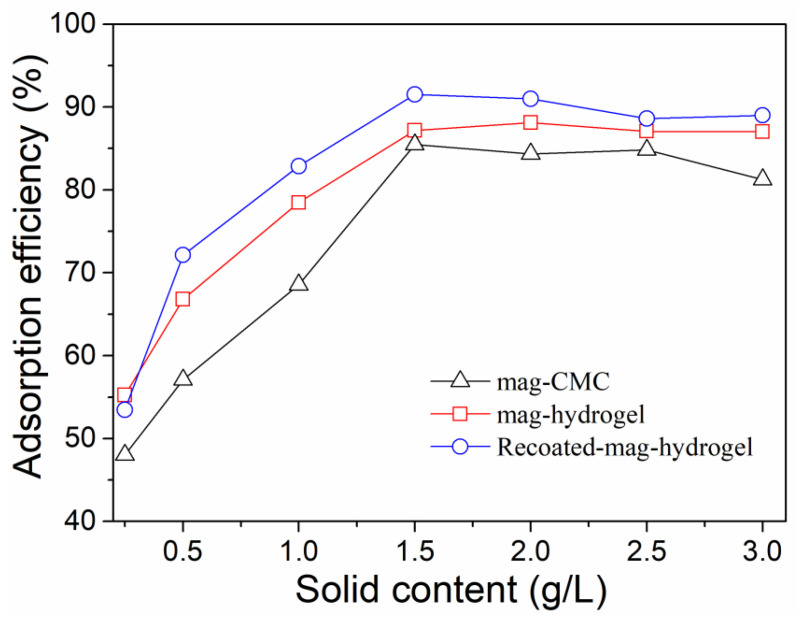
Effect of adsorbent content on MB adsorption onto magnetic nanocomposites.

Effect of adsorbent content on MB adsorption efficiency by using the present magnetic nanocomposite was studied. As illustrated in Figure 9, the adsorption efficiency of the dye was increased when the adsorbent loading amount increased, higher than 90% was obtained with 1.5 g L^−1^ adsorbent content for all three samples. The high MB adsorption efficiency resulted from the increase in the number of active adsorption sites on adsorbents to form complexes with dye. However, the adsorption efficiency of MB remained stable with the further addition of adsorbents. Higher adsorbent dose could result in the super-saturation of the suspension that lead to the collision and aggregation of magnetic nanoparticles [13]. As a result, the greatly reduced availability of surface sites would decrease the adsorption efficiency of MB dye.

### 3.4. Adsorption Isotherm

**Figure 10:**
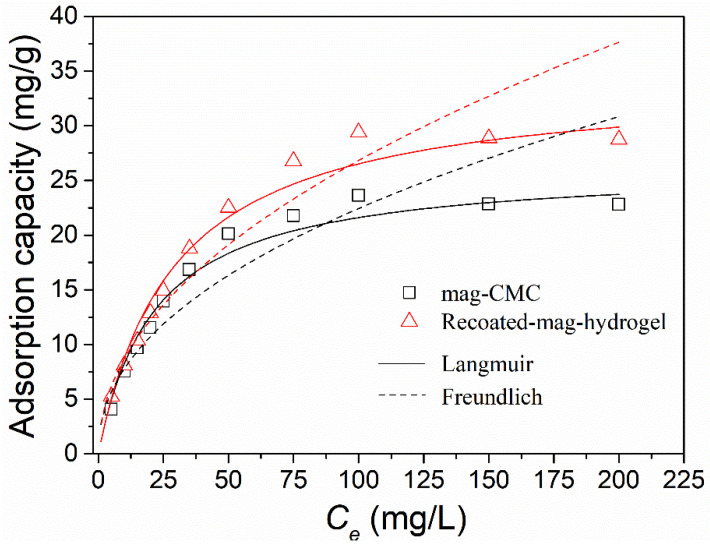
Effect of initial dye concentration on MB adsorption onto magnetic nanocomposites and fit curves of adsorption data by isotherm models.

The relationship between the adsorption capacity (*q_e_*) of magnetic nanocomposites and MB concentration (*C_e_*) at equilibrium in aqueous solution was plotted in Figure 10. The initial MB concentration ranged from 0 to 200 mg L^−1^. Obviously, the equilibrium amount of dye adsorbed increased with increasing initial dye concentration whether the magnetic nanosorbents shelled with CMC or hydrogel.

Adsorption isotherm was employed to examine the adsorption mechanisms in interaction of adsorbent with adsorbate molecules of a particular adsorption process. Langmuir (Equation (4)) and Freundlich (Equation (5)) models were two common isotherm models applied to correlate the experiment data in this work, described as follows [7]:

(4) $$q_{e}=\frac{bq_{m}C_{e}}{1+bC_{e}}$$

(5) $$q_{e}=K_{F}\times C_{e}{}^{1/n}$$

where *q_m_* (mg g^−1^) is Langmuir maximum capacity of MB on adsorbent and *b* is Langmuir constant related to adsorption energy. *K_F_* and 1/*n* are the Freundlich constants indicating the adsorption capacity and adsorption intensity of MB, respectively.

Adsorption isotherm parameters are listed in Table 1. Values of *q_m_* and *b* were calculated from intercept and slope between *C_e_*/*q_e_* and *C_e_*. Values of *K_F_* and 1/*n* were calculated from intercept and slope between log *q_e_* and log *C_e_*. It was found that the adsorption data were well fitted to Langmuir isotherm model with higher correlation coefficient (*R*^2^) of 0.99. The phenomenon suggested that the adsorption of MB on the core-shell magnetic nanocomposites was a chemical adsorption process and was regarded as monolayer adsorption. No further adsorption can be performed after the maximum adsorption capacity was reached, because a specific site can be occupied by only one molecule [37]. From Langmuir model, the maximum adsorption capacity was calculated to be 26.3 mg g^−1^ for mag-CMC, and 34.3 mg g^−1^ for Recoated-mag-hydrogel. Compared to the adsorption capacity of other adsorbents (Table 2), the maximum adsorption capacity obtained in present work enables the magnetic hydrogel nanocomposites to become competitive against other magnetic materials for MB remediation.

Besides, further analysis based on the Langmuir model, the value of separation factor constant (*R*_L_), was defined as *R*_L_ = 1/(1 + *bC*_0_). *R*_L_ had always been used to indicate that the adsorption was favorable or not. In Table 1, the *R*_L_ values were 0.098 and 0.125, which could indicate that MB adsorption by present magnetic adsorbents was favorable (0 < *R*_L_ < 1) [3].

### 3.5. Adsorption Kinetics

**Figure 11:**
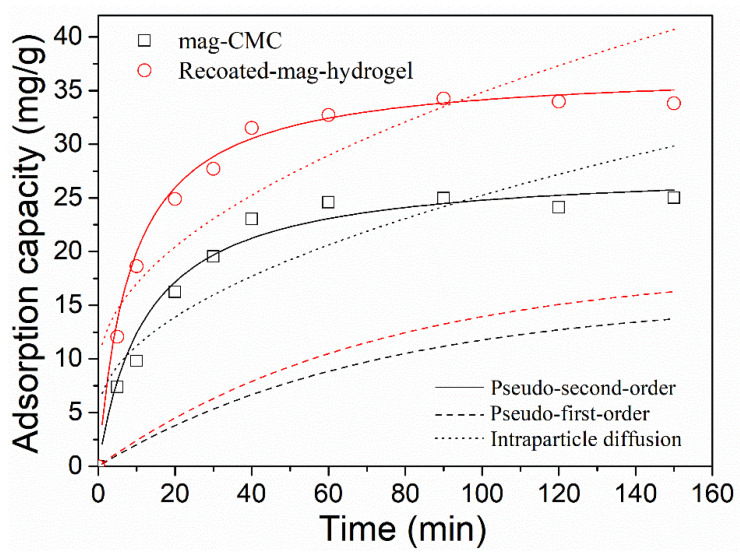
Effect of contact time on MB adsorption onto magnetic nanocomposites and fit curves of adsorption data by kinetic models.

Figure 11 displays the effect of contact time on MB adsorption onto magnetic nanocomposites. In Figure 11, the adsorption of MB increased rapidly at first 20 min and then decreased gradually, and finally, reached equilibrium at around 120 min for both of the two adsorbents. 

For determining the controlling mechanisms during the adsorption process, Pseudo-first-order in Equation (6), Pseudo-second-order in Equation (7) and Intraparticle diffusion in Equation (8) kinetic models were considered to study the adsorption rate and rate-determining step [1]:

(6) $$\log\left(q_{e}-q_{t}\right)=logq_{e}-\frac{k_{1}}{2.303}t$$

(7) $$\frac{t}{q_{t}}=\frac{1}{k_{2}q_{e}{}^{2}}+\frac{1}{q_{e}}t$$

(8) $$q_{t}=k_{i}t^{0.5}+C$$

where *k*_1_ (min^−1^), *k*_2_ (g mg^−1^min^−1^) and *k_i_* (mg g^−1^min^−0.5^) are the adsorption rate constants of Pseudo-first-order, Pseudo-second-order, and Intraparticle diffusion, respectively. Slope and intercept of experimental lines between log(*q_e_*−*q_t_*) and *t*, *t*/*q_t_* and *t*, *q_t_* and *t*^0.5^ respectively give values of *k*_1_, *k*_2_, and *k_i_*.

The adsorption kinetic parameters are listed in Table 3. From Intraparticle diffusion model, the *C* value is not 0 and the straight lines in Figure 11 do not pass through the origin, which means that in addition to the rate-limiting step by Intraparticle diffusion, other steps influence the adsorption process [44,45].

It was found that the adsorption data were well followed to Pseudo-second-order model with higher *R*^2^ of 0.99. Besides, the theoretical calculated *q_e_*_,cal_ in Pseudo-second-order model gives a better agreement with the experimental data *q_e_*_,exp_. The phenomenon demonstrated that the rate controlling process of the magnetic nanocomposites was limited by chemisorption rather than physical interaction or mass transport, similar results were reported in the literature [3,46]. More specifically, surface complexes would be formed because of the chemisorption of MB on magnetic. In Table 3, *k*_2_ was found to be higher in Recoated-mag-hydrogel than in mag-CMC, suggesting that the magnetic adsorbents with hydrogel shell had a faster adsorption of MB. The reason might be that in the adsorption process the abundant channels and pores in porous hydrogel structure would lead to higher degree of dye distribution over its surface.

### 3.6. Comparison of Mag-Hydrogel with Mag-CMC

**Figure 12:**
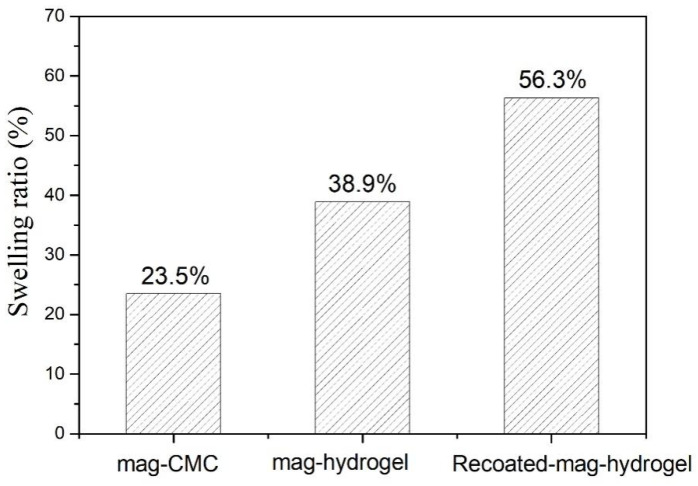
The equilibrium swelling ratio of magnetic nanocomposites (pH = 7, 25 °C).

It was found that the maximum adsorption capacity of mag-hydrogel was considerably higher than that of mag-CMC nanocomposite. In addition, recoating hydrogel shell outside the surface of Fe_3_O_4_ core would also efficiently increase the MB adsorption. Similar changes also occurred in the swelling ratios as shown in Figure 12. Therefore, it can be concluded that the surface property of the adsorbent was the main factor that can control the mass transfer rate [26]. However, the specific surface area of as-prepared mag-CMC (87.2 m^2^ g^−1^) was higher than that of mag-hydrogel (73.5 m^2^ g^−1^). This means that the adsorption capacity difference was not induced by the specific surface area. Alternatively, the surface-linked hydrogel moieties played a critical part in promoting the adsorption capacity toward MB. Firstly, CMC-g-p(AA-co-AM) moieties could provide plentiful active groups in its structure, which was expected to be beneficial for binding of dye, including the electrostatic interaction resulted from the exchange or sharing of electrons between the anionic hydrogel and cationic dyes. Secondly, compared with overlapping of CMC chains on the magnetic cores, abundant channels and pores were existed in the 3D polymer framework of swollen hydrogel, which might increase the affinity to contaminants and promote dye diffusion through the adsorbent pores (Figure S1).

### 3.7. Adsorption Mechanism

**Figure 13:**
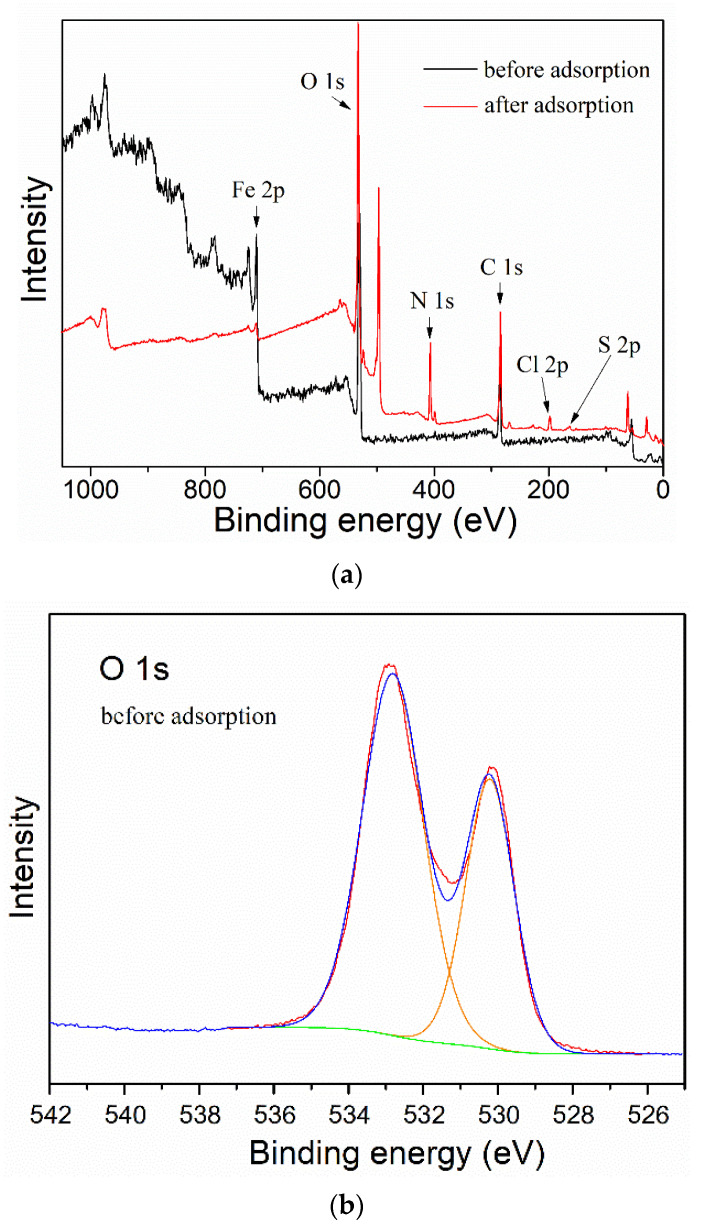

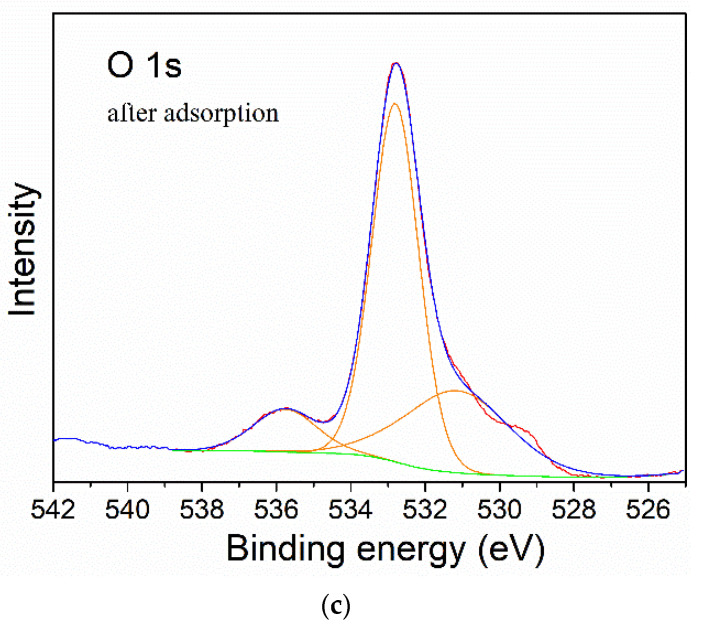
The XPS survey spectra (**a**), curve fitting for O 1s spectra of mag-hydrogel before (**b**) and after (**c**) MB adsorption.

**Figure 14:**
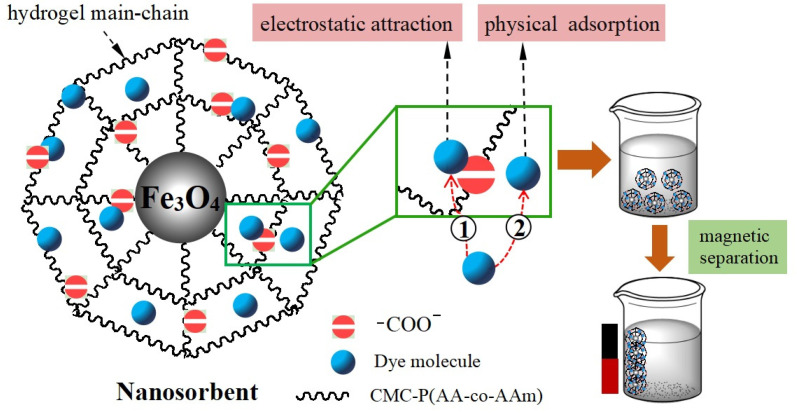
Schematic diagram of proposed interactions between mag-hydrogel and MB dye.

Finally, in order to study the attachment mechanism of MB on the mag-hydrogel, the mag-hydrogel before and after MB adsorption was investigated by XPS techniques. As depicted in Figure 13a, in addition to the characteristic peaks of C 1s (285.1 eV), O 1s (532.8 eV), Fe 2p (710.9 eV), S 2p (164.7 eV), Cl 2p (198.9 eV), and N 1s (407.3 eV) peaks clearly appeared for the mag-hydrogel after adsorption, suggesting that the MB molecules were adsorbed on the mag-hydrogel. Figure 13b,c illustrate the deconvolution of O 1s peak of mag-hydrogel with and without adsorbed MB. The two peaks observed at 532.79 and 532.81 eV were due to the O in carboxylate or carbonyl groups (O–C=O, C=O) [47]. Before adsorption, the peaks of O 1s at 530.2 eV was possibly ascribed to the Fe–O–C and/or Fe–O bond. This possibility was explained by the fact that the metal–O–C bonds formed in composite nanoparticles would shift to a somewhat higher binding energy from the metal-O bonds of metal oxides, and the characteristic peaks of the lattice oxygen in Fe_3_O_4_ (Fe–O) were reported at lower than 530 eV [48,49,50]. After adsorption of MB, the Fe–O–C and/or Fe–O bond was shifted to higher binding energy at around 531.2 eV, besides, the C=O/Fe–O–C area ratio was found to reduce after adsorption of MB, these results might be related to the electrostatic attraction between –COO^−^ groups of the hydrogel and NR_4_^+^ of MB [51]. Moreover, the considerable specific surface inside the porous hydrogel allowed the dye molecules physically-adsorbed in the narrow channels of the hydrogel internal structure. As result, double effects, including electrostatic attraction and physical adsorption, might control the adsorption properties of the mag-hydrogels. Figure 14 illustrates the schematic diagram of the proposed interactions between mag-hydrogel and MB dye. 

### 3.8. Reusability

**Figure 15:**
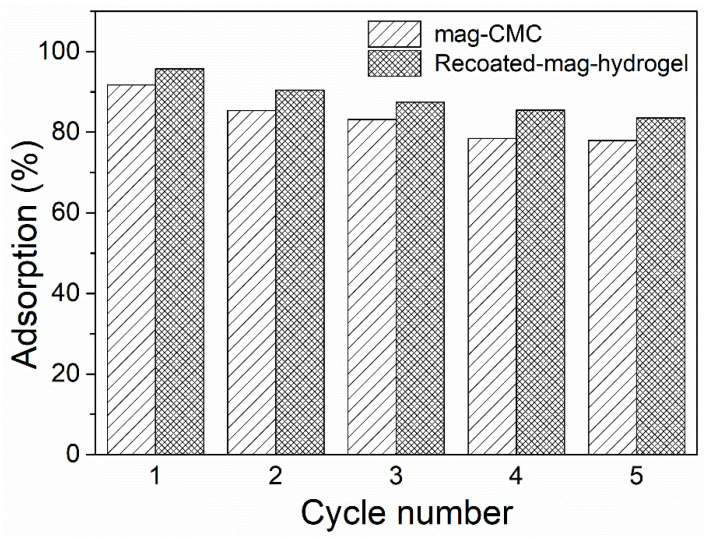
Reusability of magnetic nanocomposites in MB adsorption.

Excellent regeneration and reusability are the characteristics of an efficient adsorbent. In present study, adsorption-desorption experiments were repeated five cycles to evaluate the reusability of the hydrogel shelled magnetic nanosorbents, and in desorption tests, a low concentration of acidic solution was needed to efficiently desorb the dye molecules from MB loaded mag-hydrogel. The obtained re-adsorption efficiency is shown in Figure 15. As mentioned above, the adsorption process was a chemical reaction, thus the adsorbate cannot be completely desorbed from the adsorbent. As the cycle time increased, the MB adsorption percentage slightly decreased from 95.6% to 83.5%. The decline of MB adsorption percentage might be due to the difficulties of MB molecules diffusion form hydrogel pores. Nevertheless, the experimental results herein showed that the synthesized hydrogel shelled magnetic nanocomposite had favorable reusability property for the removal of MB from contaminated water.

## 4. Conclusions

CMC-based hydrogel nanocomposite with magnetic cores of Fe_3_O_4_ nanoparticles was successfully fabricated via the combination of graft co-polymerization and chemical co-precipitation methods. FTIR and XRD results demonstrated the incorporation of Fe_3_O_4_ within hydrogel matrix. TGA revealed the mass fraction of hydrogel coating was about 20.3%, and saturation magnetization of 39.4 emu g^−1^ as evidenced by VSM was high enough to achieve efficient separation. The magnetic nanocomposites performed well in removal dye contaminants and had maximum adsorption capacity of 34.3 mg g^−1^ at neutral pH. Adsorption isotherm data fitted well with Langmuir adsorption isotherm, indicating that the process is a chemical monolayer adsorption. Adsorption kinetic followed pseudo-second-order model, showing that the rate was controlled by chemisorption and surface adsorption was the rate-limiting step. Moreover, the prepared hydrogel shelled magnetic nanocomposite had good reusability of 83.5% after five cycles and could be used as an economic and effective adsorbents for dye pollutant removal from wastewater.

## Figures and Tables

**Table 1:** Adsorption isotherm parameters for MB adsorption.

Isotherm Models	Parameters	Mag-CMC	Recoated-Mag-Hydrogel
Langmuir	*q_m_* (mg g^−1^)	26.32	34.25
	*b* (L mg^−1^)	0.046	0.035
	*R* ^2^	0.991	0.989
	*R*_L_	0.098	0.125
Freundlich	1/*n*	0.459	0.489
	*K_F_* (mg g^−1^)(L mg^−1^)^1/*n*^	2.710	2.826
	*R* ^2^	0.877	0.933

**Table 2:** Comparison of adsorption capacity of different adsorbents for the adsorption of MB.

Materials	Experimental Conditions	*q*_max_ (mg/g)	References
magnetite/silica/pectin NPs	pH = 8, T = 298 k	178.57	[38]
HA-Fe_3_O_4_	pH = 11, T = 293 k	93.07	[39]
Fe_3_O_4_@PAH/GO−COOH	pH = 7, T = 298 k	35.96	[40]
Fe_3_O_4_-CMC-g-p(AA-co-AM)	pH = 7, T = 298 k	34.3	This work
Halloysite-TiO_2_-Fe_3_O_4_	pH = 7, T = 298 k	27.27	[41]
Fe_3_O_4_@C	pH = 7, T = 308 k	19.13	[42]
Fe_3_O_4_@NiSiO_3_	pH = 7, T = 300 k	18.11	[43]

**Table 3:** Adsorption kinetic parameters for the adsorption for MB adsorption.

Kinetic Models	Parameters	Mag-CMC	Recoated-Mag-Hydrogel
Pseudo-first-order	*q_e_*_,exp_ (mg g^−1^)	24.98	34.26
	*q_e_*_,cal_ (mg g^−1^)	15.70	18.61
	*k*_1_ (min^−1^)	0.014	0.014
	*R* ^2^	0.695	0.726
Pseudo-second-order	*q_e_*_,cal_ (mg g^−1^)	27.78	37.04
	*k*_2_ (g mg^−1^min^−1^)	0.0029	0.0032
	*R* ^2^	0.994	0.998
Intraparticle diffusion	*k_i_* (mg g^−1^min^−^^0.5^)	2.051	2.605
	*C* (mg g^−1^)	4.729	8.779
	*R* ^2^	0.803	0.772

## Data Availability

The data presented in this study are available on request from the corresponding author.

## Supplementary Materials

The following are available online at https://www.mdpi.com/article/10.3390/polym13183054/s1. Figure S1: Swelling ratio of magnetic nanocomposites (pH = 7, 25 °C).
