# Supplementary material for: Core-Shell Structured Magnetic Carboxymethyl Cellulose-Based Hydrogel Nanosorbents for Effective Adsorption of Methylene Blue from Aqueous Solution
Source: Polymers (Basel). 2021 Sep 10;13(18):3054. doi: 10.3390/polym13183054 (PMC8466880; doi:10.3390/polym13183054)
Supplement: Supplementary file 1 [file polymers-13-03054-s001.zip › polymers-1360843-supplementary.pdf]

Supplementary materials:

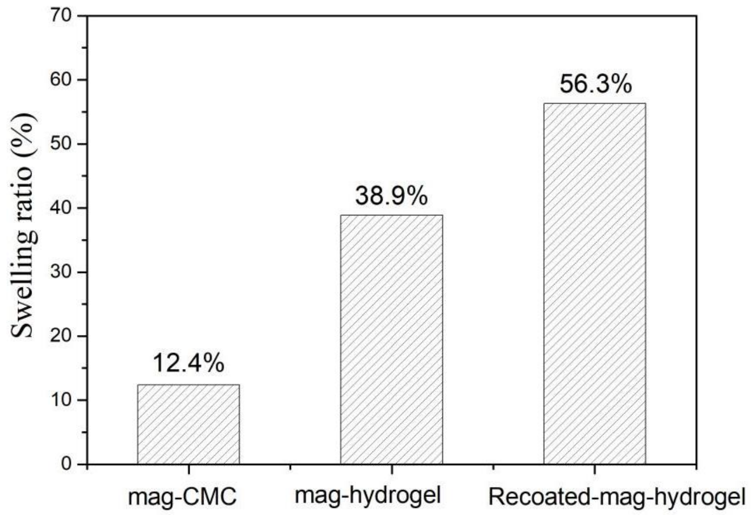

Figure S1. Swelling ratio of magnetic nanocomposites (pH=7, 25ºC).

The equilibrium swelling ratiowas calculated by the following equation:

$$\text{Swelling ratio (\%)} = (W_s - W_d)/W_d \times 100\%$$

Where W_d (g) and W_s (g) are the mass of dried and swollen samples, respectively.
